# Supplementary material for: COZOID: contact zone identifier for visual analysis of protein-protein interactions
Source: BMC Bioinformatics. 2018 Apr 6;19:125. doi: 10.1186/s12859-018-2113-6 (PMC5889581; doi:10.1186/s12859-018-2113-6)
Supplement: Supplementary file 2 — Software build. Executable binary file of the software tool. (ZIP 118,784 kb) [file 12859_2018_2113_MOESM2_ESM.zip › cozoid/platform/docs/testng-6.5.1-javadoc.zip!/org/testng/xml/package-frame.html]

org.testng.xml (TestNG)


org.testng.xml

Classes 
  
XmlClass
  
XmlInclude
  
XmlMethodSelector
  
XmlPackage
  
XmlSuite
  
XmlTest
  
XmlUtils
